# Acid-mediated Lipinski’s second rule: application to drug design and targeting in cancer

**DOI:** 10.1007/s00249-014-0953-1

**Published:** 2014-04-01

**Authors:** Ziad Omran, Cyril Rauch

**Affiliations:** 1 School of Veterinary Medicine and Science, University of Nottingham, Sutton Bonington Campus, College Road, Sutton Bonington, Leicestershire, LE12 5RD UK; 2 College of Pharmacy, Umm Al-Qura University, Al-Abidiyya, Makkah, 21955 Kingdom of Saudi Arabia

**Keywords:** Drug delivery, Pharmacokinetic, Membrane biophysics, Cancer, Warburg effect, Multi-drug resistance

## Abstract

With a predicted 382.4 per 100,000 people expected to suffer from some form of malignant neoplasm by 2015, and a current death toll of 1 out of 8 deaths worldwide, improving treatment and/or drug design is an essential focus of cancer research. Multi-drug resistance is the leading cause of chemotherapeutic failure, and delivery of anticancer drugs to the inside of cancerous cells is another major challenge. Fifteen years ago, in a completely different field in which improving drug delivery is the objective, the bioavailability of oral compounds, Christopher Lipinski formulated some rules that are still used by the pharmaceutical industry as rules of thumb to improve drug delivery to their target. Although Lipinski’s rules were not formulated to improve delivery of antineoplastic drugs to the inside of cancer cells, it is interesting to note that the problems are similar. On the basis of the strong similarity between the fields, we discuss how they can be connected and how new drug targets can be defined in cancer.

## Lipinski’s rules applied to drug bioavailability

The 90s were gloomy years for the pharmaceutical industry with productivity falling below expectations. Indeed, the ten leading companies’ newly marketed compounds increased their revenues by only ~10 %, and the average innovation deficit was ~1.3–1.8 new chemical entities per year (Drews 2003). As the time from drug discovery to launch is currently ~12 years and costs ~$750 million/drug, the pharmaceutical industry is determined to reduce both the cost and time scale of this process; it is, therefore, understandable that the strategies adopted by these companies are those which provide information in advance of costly clinical trials. A significant obstacle to this is determining the properties of a drug that facilitate its delivery to, and uptake by, target tissues and/or cells to avoid unsuccessful but nonetheless expensive clinical trials.

The bioavailability of drugs and hence their ability to interact with their targets can be summarized by four notions grouped under the acronym ADME, which stands for absorption, distribution, metabolism, and excretion. Each of these notions involves a particular aspect of the physiological interactions between body tissues and drugs, which explain drug bioavailability.

To circumvent the inherent difficulty linked to ADME-related problems, Lipinski and collaborators produced a set of rules to identify the optimum physicochemical properties required for an oral compound to achieve maximum bioavailability, i.e. to cross all biological barriers before reaching its target. They studied all marketed drugs and deduced similarities or common important properties of all the different active compounds. In this context they formulated a set four rules known, today, as Lipinski’s rules.

The first rule is based on the lipophilic index of drugs (octanol–water partitioning: log *P* < 5). The second rule is based on the drugs’ molecular weight (MW), which must be <500. The third and fourth rules are based on the nature of the charge on the drugs (number of hydrogen-bond donors, i.e. number of OH + NH bonds <5; and number of hydrogen bond acceptors, i.e. number of O + N atoms <10). Together, these rules define the 90th percentile of the physicochemical properties drugs should have to achieve the greatest bioavailability (Lipinski et al. 2001). Because these rules were formulated for synthetic chemicals, they were initially criticized, because many drugs are natural compounds; it was later found, however, that natural compounds, unsurprisingly, also follow Lipinski’s rules (Quinn et al. 2008). These rules are now established models for drug discovery and have been largely embraced by the pharmaceutical industry. However, a full and systematic scientific investigation of the way drugs interact with cells or tissues to generate these rules is still in its infancy and has yet to be fully conducted.

What is remarkable however is that although these rules may involve macro complex systems (the body), they seem to be equally important when single cells are considered.

### Lipinski’s rules applied to drug entry into cells

Of the four rules, the second (MW <500) stands out because of its apparent simplicity, being unrelated to the complex physicochemical properties of a drug (as are its charge state or lipophilicity) but governed solely by a drug’s size or volume. In addition, although bioavailability is often considered in terms of biochemical interaction, the MW does not involve such interactions because it is not implicated in defining affinity between chemicals.

When physicists or biophysicists consider the MW of chemicals they consider the size or volume of the chemical. In physics, volume is important because it helps to define pressure, i.e. force per unit surface area. Said differently, if the MW of a chemical is involved in Lipinski’s second rule it is because pressure must be present so MW is an important property. To be bioavailable, drugs must traverse cellular barriers (usually epithelia), and to traverse cellular barriers drugs must cross lipid membranes. It is natural to believe the MW of chemicals is important because of the surface pressure that exists in bilayer membranes. Naturally, this conclusion is true only if chemicals are not diffusing across the membrane via specific membrane pores (e.g. aquaporins).

The membrane surface pressure results from optimization of the energies involved in lipid–lipid interactions (Rauch 2009b). Many different lipids form the membrane and the cell uses much energy conserving the important heterogeneity involved in membrane integrity. Two main types of energy are involved in systems composed of lipids, one linked to the attraction between lipids and the other linked to the repulsion between them. The source of attraction between lipids is related to their aliphatic chains, which have no affinity for water and, as a result, lipids will do their best to avoid increasing their free surface area in the membrane, to minimize contact with water. The source of repulsion, however, is linked to electrostatic or steric repulsion involving polar momentum, electrostatic charges of the lipid hard core that will try to increase the free surface area per lipid. In soft systems, for example cell membranes, there is no frustration linked to uncompensated energy. This means that the system tries to be in a minimum energy state. The minimum energy state for a bilayer membrane is defined by the optimum cross-sectional area per membrane lipid, taking into consideration the aforementioned repulsion and attraction (Annexes 1 and 2).

Incorporation of any drug into a lipid bilayer membrane will, therefore, perturb the minimum energy state of the membrane by forcing lipids into closer contact––i.e. by forcing the packing of the lipids. As a response, the membrane will try to expel the drug from the lipid phase to re-establish the equilibrium. It is now clear that the larger the drug the greater the perturbation of the membrane. As a result lipids will apply a force against entry of drugs into the membrane that is necessarily a function of their size. In this context, a sort of Lipinski’s second rule concerning the molecular weight of drugs can be applied at the cellular level.

**Fig. 1:**
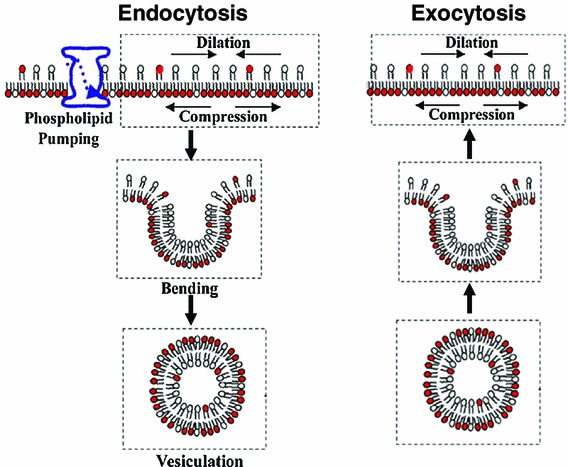
The lipid number asymmetry-induced fluid phase endocytosis model. Schematic diagram of the current model applied to living cells which links fluid phase endocytosis and membrane phospholipid number asymmetry maintained by a flippase. In the *left figure*, the translocation of dark-head lipids into the inner leaflet induces differential lipid packing between leaflets (different surface tension) leading to membrane bending and vesiculation (Farge et al. 1999; Rauch and Farge 2000b). Note that it is assumed that the membrane recycling that occurs in cells, i.e. the exocytosis of vesicles of a size similar to endocytic vesicles, also enables maintenance of lipid asymmetry at the level of the plasmalemma. The relationship between lipid number asymmetry and the vesicle radius is given by $R = 8k_{c} /h\Delta \sigma$ or, equivalently, $R = 4k_{c} /hK \cdot 1/(\delta N/N_{0} )$, where $k_{c}$, $K$, $h$, $\Delta \sigma$ and $\delta N/N_{0}$ are the membrane bending modulus, membrane elastic modulus, membrane thickness, surface tension difference, and the lipid number asymmetry between leaflets. Accordingly, lipid number asymmetry has been experimentally deduced from studies on cells for which $\delta N/N_{0} = 2 \pm 0.5\%$ providing a ~35 nm vesicle radius (Rauch and Farge 2000b)

One thing which must be clarified, however, is that a membrane is not randomly composed. Some lipids are more abundant on one leaflet than on the other, which creates dissymmetry (Seigneuret and Devaux 1984). Furthermore, packing of lipids on each leaflet (i.e. the surface pressure of each leaflet) is not the same on the outer and inner leaflets. The surface pressure of the inner leaflet is slightly more important than that of the outer leaflet, which is involved in endocytosis (Fig. 1) (Rauch and Farge 2000a).

**Fig. 2:**
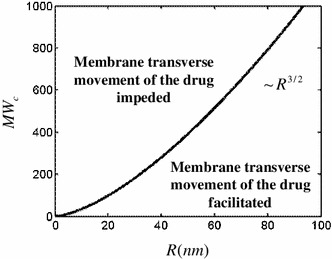
**a** Relationship between drugs’ MW and their ability to bypass the membrane barrier as a function of vesicle radius $R\text{ }({\text{nm}})$ expressed in nanometers, scaling as ${\text{MW}}_{c} \sim R^{3/2}$ (exactly: ${\text{MW}}_{c} \cong 1.1\;R^{3/2}$using constants seen in the text)

 Together, the outer and inner leaflets create a perfect barrier to drugs. In this context it is possible to define what would be the theoretical MW limit (details are given in Annex 1):

1 $$ {\text{MW}}_{c} = (4/3\sqrt \pi )(hRk_{B} T/8k_{c} )^{3/2} $$

where, $k_{B}$ is Boltzmann’s constant, *T* the temperature in Kelvin, $R$ the vesicular radius, $h$ the membrane thickness, and $k_{c}$ the membrane bending modulus. This equation provides a law with regard to drug size (or MW) selectivity for permeation across cellular membranes. Use of the numerical values of physical constants and biological properties reveals that ${\text{MW}}_{c} \cong$ 250–500 at 37 °C (Rauch and Pluen 2007). The larger value of this range is remarkably close to Lipinski’s second rule. A representation of Eq. 1 is given in Fig. 2.

In this context of mechanical filtration of drugs on the basis of their size, the cell has found ways of modulating entry of drugs. An interesting case is cancer, in which the pH gradient across the membrane can drive or control the influx of drugs.

### Lipinski’s rules applied to drug entry into cancer cells, for which pH is an important condition

Cancer cells are exquisitely difficult to control and, ultimately, to kill. There are many reasons for this; one of direct interest is the notion that entry of drugs into cells (i.e. Lipinski’s rule for cells) seems to be linked to the pH gradient across the cell membrane.

A crucial event (cause or effect) in the transformation of normal cells into cancerous cells was discovered in 1924 by Otto Warburg, who first described a switch of metabolism (i.e. cellular respiration) to glycolysis (Tennant et al. 2009) despite the relative inefficiency of this process for creation of adenosine triphosphate (ATP). Today it is well acknowledged that heterogeneous tumor cancer cells organize themselves to use either oxidative or glycolytic metabolism or both, thereby promoting strong survival ability (Porporato et al. 2011). A direct consequence of cancer cells-specific metabolism is a shift in pH, in part associated with the creation of lactate (and hydrogen) an end-point of the glycolytic metabolism. Further studies have demonstrated that the alkalinization of the intracellular pH (pHi) of cancer cells is accompanied by acidification of the extracellular environment (pHe) (Schornack and Gillies 2003), because of the activity of proton pumps including vacuolar-type ATPase (V-ATPase), the proton transporters Na^+^/H^+^ exchanger (NHE), the monocarboxylate transporters (MCT), the bicarbonate transporter (BCT), the carbonic anhydrases, ATP synthase, and the Cl^−^/HCO_3_ exchanger (Daniel et al. 2013).

The pH gradient phenomenon is now believed to be involved in both post-transformation of the neoplastic phenotype and activation and etiopathogenesis of the metastatic process (Harguindey et al. 2005, 2009; Reshkin et al. 2000, 2012).

**Fig. 3:**
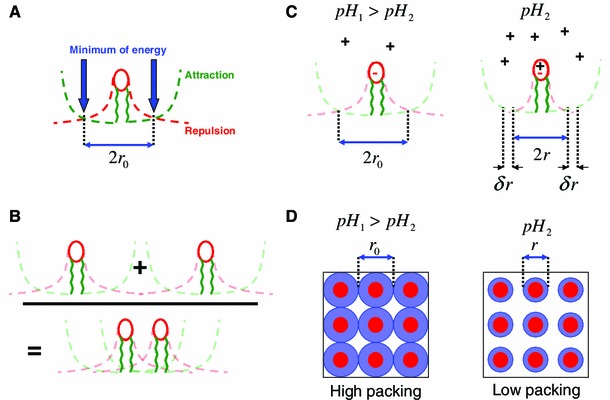
Effect of pH on the packing of lipids. **a** Assuming a leaflet composed of charged lipids. The optimum area per lipid is determined by the competition between energy that reflects lipids attraction linked to their hydrophobic tails and repulsion energy which we will assume to be linked to a net charge carried by all lipids. The competition between these two terms defines an energy minimum. Note that in the figures *r*_0_ corresponds to the optimum distance between adjacent lipid heads. **b** Thus the minimum energy determines the optimum distance between lipids, including their optimum area in the monolayer. Note that the packing of lipids is not always defined by physical contact and that, accordingly, there is room to change this packing. **c** With regard to negatively charged lipids, an increase in the concentration of hydrogen ions enables more hydrogen ions to interact with lipids’ heads. Thus, by masking their negative charge, the long-range repulsion between lipids is disturbed. The resulting effect will be alteration of the positioning of the energy minimum, so the lipids become closer. **d** Top view of a portion of the membrane. The lipid’s head is colored in *red* and the optimum area per lipid driven by repulsive and/or attractive interactions is drawn in *blue*. Changes in pH are expected to redefine the optimum area per lipid, and thus their packing. In the figure a decrease in the pH is represented, i.e. pH_2_ < pH_1_. In conclusion, a low cytosolic pH is expected to reduce the surface area per lipid. Lipids should have more room, thus reducing their packing. Changing the cytosolic pH is thus expected to affect the packing of inner leaflet lipids because it is in this leaflet that negatively charged lipids are found. To conclude, the packing of lipids can vary even though the number of lipids is unchanged. In this case, pH-driven alteration of lipid repulsion causes this change. Accordingly, this change is expected to affect the transverse movement of drugs across the membrane and thus their efficacy, as demonstrated by Rauch (2009b)

With regard to membrane permeability, pH is an important condition because it is related to the concentration of free hydrogen ions, which can affect electrostatic interactions between lipids. Because some lipids from the inner leaflet (e.g. phosphoinositides, phosphatidylserine, and phosphatidic acid) bear a negative charge, they can interact weakly with hydrogen ions, resulting in less repulsion between them (Fig. 3) (details are given in Annex 2). As a result, pH can cause changes of the surface pressure of lipid leaflets and affect the permeability of the resulting membrane to drugs, assuming some lipids are negatively charged and interact with hydrogen. In this context it has also been noted that the pH gradient mentioned above is a driver of drug resistance in cancer (Rauch 2009b) and that, irrespective of such drug transporters as *P*-glycoprotein, the size of drug is an important physical property in drug resistance (Rauch 2009a). In this context, the accumulation of anticancer alkaloid drugs inside lysosomes often observed in MDR cancer cells results from drugs being mechanically trapped at the membrane level and internalized via endocytosis. A change in pH gradient, for example via use of proton pump inhibitors, would improve drug delivery inside cells.

It follows here that a better understanding of Lipinski’s rules enables one to comprehend why pH regulation in cancer is so important and why it is a good target for modulating drug entry into cancer cells. Indeed, pH abnormalities in cancer not only modify the charge of weak acids and bases (hence their octanol–water partitioning) and their ability to interact with the membrane lipid phase, they also act on the fluidity of lipid bilayers and therefore on the ability of drugs to directly cross membranes.

### Lipinski’s rules in cancer cells modulated by proton pump inhibitors (PPIs)

Because the pH gradient is important to drug resistance in cancer and affects membrane permeability, and because tumoral extracellular pH is low, as a result of cancer cell metabolism, it may be possible to target pH to control the delivery of chemicals into the tumor.

**Fig. 4:**
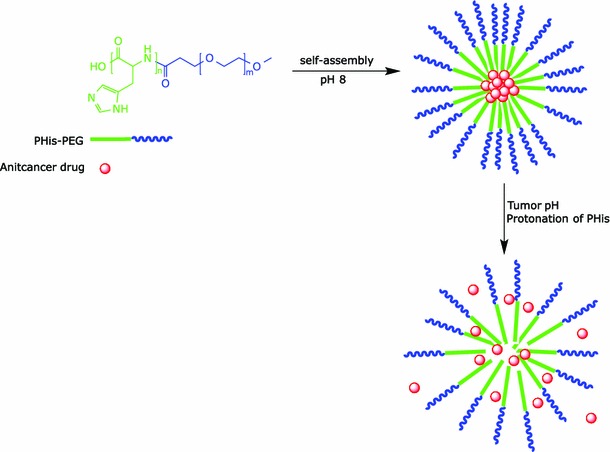
Acid-induced drug release from pH-responsive micelles

Two main strategies have been developed to target the relatively acidic extracellular microenvironment of the tumor. First is the development of biologically inert prodrugs of the anticancer agents that will release the cytotoxic entities under the effect of the low pH. Examples of pH-sensitive protecting groups that have been used to mask anticancer drug activity include imine, hydrazone, carboxylic hydrazone, ketal, acetal, *cis*-aconityl, and trityl (Binauld and Stenzel 2013). The second approach is to load the anticancer drug into pH-responsive nanocarriers. Such nanocarriers are designed to be stable under neutral physiological pH, and to collapse under slightly acidic pH, releasing the entrapped cytotoxic agent within the tumor tissue, followed by enhanced drug uptake by cancer cells because of high concentration gradients, while maintaining a low rate of release during circulation in the blood (Shen et al. 2008). For example, Lee et al. (2003a) developed pH-destabilizable poly(l-histidine)-*b*-poly(ethylene glycol) (abbreviated as PHis-PEG)-based micelles. The water-solubility of PHis is pH-dependent, as a result of protonation of its imidazole *sp* ^2^ nitrogen at acidic pH (Fig. 4). The critical micelle concentration (CMC) at pH 8.0 was 2.3 mg/l. The CMC increased markedly on reducing the pH. Micelles prepared at pH 8.0 were gradually destabilized below pH 7.4, and no micelles could be detected below pH 5. This increase in CMC at lower pH is caused by protonation of the imidazole ring; it leads to reduction of its hydrophobicity and increased water solubility of the copolymer (Lee et al. 2003b). Loading of such pH-responsive micelles with doxorubicin (DOX) increased its in-vivo plasma half-life (*t*_1/2_) and its area under the concentration curve (AUC) more than fivefold. Similarly, DOX-loaded micelles significantly increased inhibition of the growth of A2780 xenografts in nude mice after i.v. administration compared with free DOX treatment. The volume of tumors treated with the pH-sensitive micelles was approximately a factor of 4.71 smaller than those treated with free DOX after 39 days (Gao et al. 2005). As a result, it is possible today to target tissues where the surrounding pH is low.

DOX is a weak base; once protonated it cannot traverse the membrane. The effect of cancer pH on DOX efficacy has already been extensively studied (Altan et al. 1998; Rauch 2009b). DOX efficacy can be increased by use of appropriate pH-sensitive micelles; it can also be improved by targeting pH regulation by cancer cells and, in particular, their ability to release protons. Dual loading of micelles with both proton-pump inhibitors (PPIs) and DOX may, in fact, increase the efficacy of DOX in the short term. Indeed, PPIs would acidify the cytosol, making the membrane more fluid with regard to DOX and, at the same time, DOX would be released by the micelles to act on its target. Naturally, a change in pH (alkalization) of the extracellular environment linked to the activity of the PPIs would reform the micelles enabling them to keep their unused load for later. Under these conditions, loading micelles with DOX and PPIs (or other inhibitors of pH regulators, for example NHE, MCT, or BCT) can be considered as a new potential strategy against cancer.

## Conclusion

Studies highlighting the membrane as a biomechanical object date from the 1970s (Sheetz et al. 1976; Sheetz and Singer 1974). Since then, much effort has been devoted to investigation of the effects of these biomechanical properties on basic biology. Warburg’s discovery in the 50s demonstrated the importance of pH in cancer; its importance in multidrug resistance has been revealed more recently. In 2001, Lipinski et al. demonstrated the effect of oral drug MW on delivery. There are clear overlaps between these research fields that require full investigation. Being able to harness the delivery of chemicals is still an outstanding challenge for the pharmaceutical industry and cancer research and it is hoped that interaction between these research fields and biophysics will lead to new ways of controlling the delivery of chemicals.

## Annex 1: effect of membrane physical properties on drug permeation

To traverse cellular barriers, drugs must cross lipid membranes. For this, Lipinski’s second rule postulates that drugs must have a MW <500. Therefore, in the sum of energies making up the total activation energy required for a drug to cross cellular membranes, there must be an energy term that is a specific function of the drug’s dimensions so that the drug–membrane interaction yields an energy ${\ge}k_{B} T$ (where* k*_*B*_ is Boltzmann’s constant and *T* the temperature in Kelvin). In this case, i.e. when the plasma membrane is considered, the physical property that best fits such an interaction is the leaflets’ surface pressure, $\sigma$. In cells, however, two types of membrane tension can be distinguished, the mean surface tension denoted $\sigma_{0}$, which corresponds to the sum of individual leaflet’s surface tension, and the difference between surface tensions $\Delta \sigma$, which corresponds to the difference between an individual leaflet’s surface tensions, i.e. those of the inner and outer leaflet. However, cells have a large membrane reservoir and an average membrane tension that is remarkably low, $\left| {\sigma_{0} } \right|\sim 10^{ - 2} - 10^{ - 3}\,{\text{mN/m}}$ (Hochmuth et al. 1996; Raucher and Sheetz 1999) compared with the magnitude of the difference in surface tensions between leaflets, $\left| {\Delta \sigma } \right|\sim 0.9\text{ }{\text{mN/m}}$ (Rauch and Farge 2000b). Accordingly and given the magnitude of this property, $\Delta \sigma$ is more likely to be involved in impairing the transverse movement of chemicals. Dimensionally speaking, it follows that the magnitude of the drug’s critical cross section, $a_{c}$, can be defined by:

2 $$ a_{c} = - k_{B} T/\Delta \sigma $$

In Eq. 2, the minus sign indicates that the membrane is compressed when drugs traverse it. The surface tension difference, $\Delta \sigma$, is associated with the effects of lipid flippases, which maintain membrane lipid asymmetry (Seigneuret and Devaux 1984). In particular, it has been demonstrated that a particular membrane flippase actively relocates phosphatidylserine (PS) and phosphatidylethanolamine (PE) from the outer into the inner leaflet of the cell membrane. One consequence of this inward pumping is a constantly more highly packed inner leaflet, because it contains more phospholipids than the outer leaflet (Fig. 1). It has been demonstrated that this lipid packing asymmetry between the membrane leaflets leads to fluid phase endocytosis (Devaux 2000; Farge 1995; Farge et al. 1999; Rauch and Farge 2000b) and that the vesicle radius, $R$, can be expressed as (Fig. 1a) (Rauch and Farge 2000b):

3 $$ R = - 8k_{c} /h\Delta \sigma $$

where $k_{c}$ and $h$ are, respectively, the membrane bending modulus and membrane thickness. For small drugs their MW is proportional to their Van der Walls’ volume (expressed in $\dot{A}^{3}$), i.e. ${\text{MW}}\sim V\sim a^{3/2}$, by using Eqs. 1 and 2, a critical MW (${\text{MW}}_{c}$) can be determined (Rauch and Pluen 2007):

4 $$ {\text{MW}}_{c} = (4/3\sqrt \pi )(hRk_{B} T/8k_{c} )^{3/2} $$

Eq. 4 is Eq. 1 in the text.

## Annex 2: effect of pH on lipid–lipid interaction

To determine and model how pH alters the mechanical properties of the cell membrane we consider the thermodynamic equilibrium of an ideal leaflet, namely a surface $S$, composed of $N$ identical lipids. The optimum area per lipid in the monolayer, $a_{0}$, can be determined by optimizing the contribution of different energies arising from the structural properties of lipids, of which the main interactions are hydrophobic, steric, and electrostatic. The electrostatic interactions between lipids can be subdivided into charge–charge, charge–dipole and dipole–dipole interactions. However, the magnitudes of charge–dipole and dipole–dipole interactions are much weaker and relatively short-range compared with strong and long-range charge–charge interactions ($\gg k_{B} T$, where $k_{B}$ and $T$ are, respectively, Boltzmann’s constant and the temperature in Kelvin) (Gershel 1995). Therefore, the hydrophobic interaction will be represented by a single energy term ($E_{1}$). The membrane energy resulting from steric, charge–dipole, and dipole–dipole interactions (both short-range, relatively weak interactions $\sim k_{B} T$) will be represented by a single energy term ($E_{2}$). Finally, $E_{3}$ will characterize the charge–charge interactions ($\gg k_{B} T$). The first energy ($E_{1}$) is linked to the non-polar (hydrophobic) part of the lipids, and increases as the surface area per lipid increases (because of contact with water). As a result, this term is positive and proportional to the non optimized area per lipid, $a$, written in the form:

5 $$ E_{1} = {\text{KN}}a $$

where $K$ has the dimensions of tension (i.e. a 2D elastic modulus).

The second energy ($E_{2}$) describes short-range and weak interactions between lipids. This energy is proportional to the lipid density ($N/S$) and to the number of close neighborhoods, $z$, located in the vicinity of each lipid. Consider a 2D lattice in which each site is occupied by a lipid. In this lattice, the probability of the presence of a given lipid is $\sim (N/S)\theta^{2}$ where the characteristic length that defines the lattice mesh is $\theta$, and is expected to be numerically close to the lipid head radius, assuming a rod-like shape for lipids. As one considers weak interactions only, the interaction energy per lipid is $\sim z\tilde{\varepsilon }(N/S)\theta^{2}$, where the number of close neighborhoods is $z$ and $\tilde{\varepsilon }$ is the typical energy involved in pair-interaction between lipids. Repeating this operation for each lipid of the monolayer it follows that the total interaction energy is $\sim z\tilde{\varepsilon }(N/S)\theta^{2} N/2$, where the factor $1/2$ is present to avoid counting the same pair-interaction twice. Finally, noting $z\tilde{\varepsilon }\theta^{2} = \nu \cdot k_{B} T$, where $\nu$ is similar to the second viral coefficient of a 2D polar head gas, it follows that this energy can be written as $E_{2} = Nk_{B} T\nu /2 \cdot (N/S)$. Given that $N/S = 1/a$ it follows:

6 $$ E_{2} = \frac{1}{2}Nk_{B} T\nu \frac{1}{a} $$

The third energy of interest ($E_{3}$) is the energy between charged lipids. One will assume homogenous distribution of charged lipids in the membrane and that, because of the presence of free cytosolic electrolytes, the net charge of the lipid is screened over a critical lateral length $l_{c}$, which is Debye’s length (Nguyen et al. 2005). Note that $l_{c}$ is classically defined as the square root of the sum of squared ionic concentrations and any changes in the membrane potential, reflected by a change in ionic concentrations, would affect $l_{c}$. One will note $p_{0}$ the probability that a given lipid in the monolayer is charged, i.e. $p_{0}$ is the ratio between the number of charged lipids and the total number of lipids. Under such conditions, a given charged lipid can affect another charged lipid only if the latter is within the surface area defined by the critical length $l_{c}$ and expressed as $\pi l_{c}^{2}$ and the probability that a given charged lipid interacts with another is $p_{0} \pi l_{c}^{2} /a$, where $\pi l_{c}^{2} /a$ is the number of lipids in the surface area. It follows that the interaction energy between a given charged lipid and another in the monolayer can be written as $\bar{\varepsilon }(l_{c} )p_{0} \pi l_{c}^{2} /a$, where $\bar{\varepsilon }(l_{c} )$ is the interaction energy that is also a function of the critical length $l_{c}$. Repeating the same operation over each charged lipid composing the monolayer, without counting the same pair-interaction twice, it follows that:

7 $$ E_{3} = \frac{1}{2}N\bar{\varepsilon }(l_{c} )p_{0}^{2} \pi l_{c}^{2} \frac{1}{a} $$

In Eq. 7, a literal expression of $\bar{\varepsilon }(l_{c} )$ must be given. As a mean field approach has been considered so far, $\bar{\varepsilon }(l_{c} )$ represents the characteristic energy linked to electrostatic interactions between two charges, i.e. $\bar{\varepsilon }(l_{c} )\sim q^{2} /Dl_{c}$, where $q$ and $D$ are the monovalent lipid charge and the dielectric constant of water respectively (Nguyen et al. 2005). Assuming $\bar{\varepsilon }(l_{c} ) = \bar{\varepsilon }_{0} /l_{c}$ where $\bar{\varepsilon }_{0}$ is a function of the charge and the dielectric constant it follows that:

8 $$ E_{3} = \frac{1}{2}N\bar{\varepsilon }_{0} p_{0}^{2} \pi l_{c} \frac{1}{a} $$

Given the set of energies, the area per lipid can be optimized: $\left[ {\partial_{a} \sum\nolimits_{i = 1,2,3} {E_{i} } } \right]_{{a = a_{0} }} = 0$ and it follows that:

9 $$ a_{0}^{2} = \frac{{k_{B} T\nu }}{2K}\left[ {1 + \frac{{\bar{\varepsilon }_{0} }}{{k_{B} T}}p_{0}^{2} \frac{{\pi l_{c} }}{\nu }} \right] $$

Assuming that a hydrogen ion and a negatively charged lipid interact with energy $- e_{0}$ ($e_{0} > 0$ is the magnitude of the interaction). In this case, each negatively charged lipid can be in two states, occupied (i.e. interacting with hydrogen ion) or non-occupied (i.e. free of hydrogen ion). It follows that the partition function of a negatively charged lipid is $\zeta = 1 + e^{{\left( {e_{0} + \mu } \right)/k_{B} T}}$ ($\mu \sim k_{B} T\ln \left( {C_{{H^{ + } }} \times V_{0} /V_{{H^{ + } }} } \right)$ is the chemical potential of hydrogen ion in solution and $C_{{H^{ + } }}$, $V_{{H^{ + } }}$ and $V_{0}$ are, respectively, the volume concentration of hydrogen, the volume of an hydrogen ion, and the typical volume of ions in the cytosol). Using statistical physics, the probability that a lipid is free from hydrogen is $p_{0} = 1/\zeta$. This last relationship in conjunction with Eq. 9 provides the relationship between the free surface area per lipid and the volume concentration of ions in solution.
